# Development of a Pain Measurement Device Using 3D Printing and Electronic Air Pressure Control

**DOI:** 10.3390/biomedicines13020254

**Published:** 2025-01-21

**Authors:** José Manuel Sierra, Mª del Rocío Fernández, José Luis Cortizo, Juan Díaz González

**Affiliations:** 1 Department of Mechanical Engineering, University of Oviedo, 33203 Gijón, Spain; jmsierra@uniovi.es (J.M.S.); jcortizor@uniovi.es (J.L.C.); 2 Department of Electronic Engineering and Control Systems, University of Oviedo, 33203 Gijón, Spain; jdiazg@uniovi.es

**Keywords:** 3D printing in medicine, rapid prototyping, functional prototypes, experimental analysis

## Abstract

This article describes the design of a wireless pain monitor system, also known as a pain meter, which can be used to diagnose people with fibromyalgia. **Background/Objectives**: As the test should be done while a simultaneous Magnetic Resonance Imaging (MRI) scan is being performed on the patient to observe their brain activity, the device must not have metallic components. **Methods**: Solid modelling and additive manufacturing were used for the manufacture of the device, an electropneumatic control has also been defined, and several prototypes were manufactured and tested. The work focuses on the validation of the designed pain meter, built by Material Extrusion (MEX) technology in different materials and with different printers. The surface finishes and manufacturing tolerances of the critical parts were tested, and their suitability for the necessary functions is verified. **Conclusions**: A proper mechanical pain meter device has been designed to be used in fibromyalgia diagnosis without metal components nor wires, which is therefore compatible with simultaneous MRI.

## 1. Introduction

Numerous references to 3D printing applications in medicine can be found in the literature [1,2,3,4]. In the field of medical devices, motivated by the interest of clinicians, new applications are being designed for specific fields [5,6,7,8,9]. High competitiveness in small-scale production of complex shaped products and the facilitation of prototyping due to the ease of communication between engineers and clinicians makes 3D printing the most suitable emerging technology [10]. The 3D printing approach based on extrusion, commonly known within the industry as Fused Deposition Modeling (FDM^®^) or among additive manufacturing enthusiasts as Fused Filament Fabrication (FFF) [11], has become a popular additive manufacturing technology. In this paper, it is referred to as Material Extrusion (MEX), following the denomination of ISO/ASTM 52900 standard [12]. Advantages of this technology include the wide variety of materials available, quick material change, low maintenance costs, quick production of thin parts, a tolerance equal to +0.1 mm overall, no need for supervision, no toxic materials, very compact size, and low-temperature operation [13]. Moreover, complex shapes can be achieved, with adequate strength for medical applications, without metal parts [14]. Even today, using this technology, it is possible to integrate circuits, sensors, and actuators in a prototype made using MEX [15,16]. MEX technology is available anywhere in the world (universities, hospitals, even at home); so, if the CAD files are available, any design can be reproduced and used anywhere in the world, even in low-income countries. In this context, MEX has been selected for manufacturing of the pain meter prototypes.

Fibromyalgia is a disease that causes generalized musculoskeletal pain and a painful sensation of pressure in specific points, also known as sore spots. It causes associated pain in muscles and fibrous tissue [17]. Although it is a disease that is not talked about much, it occurs quite frequently and is suffered by between 4% and 6% of the world’s population, being more frequent in women. Currently, there are no specific laboratory or imaging tests for fibromyalgia [18].

In order to make a more specific diagnosis possible, clinical criteria of the American College of Rheumatology are followed. One of the recommended tests is known as the thumb test [19], based on the application of pressure and pinching on a thumb for a certain time. It is a simple and convenient way of both detecting the disease and measuring its degree (mild, moderate or high). This test subjectively measures the patient’s sensation of pain. Functional magnetic resonance imaging (fMRI) has been used to demonstrate changes in the brain that are consistent with chronic pain [20]. For a better diagnosis, it is proposed to relate it to the objective response observed in a simultaneous Magnetic Resonance Imaging scanner (MRI) [21,22,23].

A pain meter, or so-called dolorimeter [24], is an instrument used to measure pain threshold and pain tolerance. Dolorimetry has been defined as the measurement of pain sensitivity or pain intensity [25].

There are several types of pain meters on the market: both digital and analogue pain meters are available, though the latter are most widely used today. They have metal components so they cannot be used in an MRI machine.

**Figure 1:**
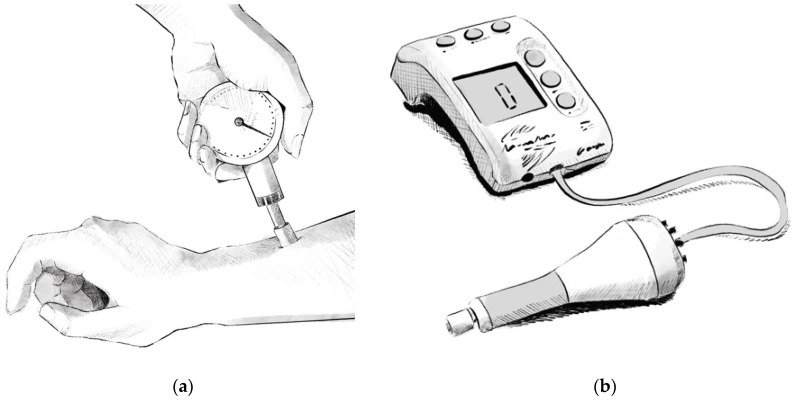
(**a**) Analogue pain meter; (**b**) digital pain meter.

The analogue pain meter (Figure 1a) is pushed over the skin, until the patient reaches maximum pain tolerance. This type of device displays the pressure that has been applied on a dial, whereas manual use makes it difficult to maintain the same pressure for a given time.

Digital pain meters (Figure 1b) are capable of collecting, storing and calculating statistics with the pressure applied. They are usually accompanied by computer software to collect the data. Other types of pain meter have been proposed [26]. Nevertheless, none of these devices are metal-free, and therefore they cannot be introduced into an MRI machine.

A characteristic of all the commercial pain meters analysed, in addition to having metal elements, is that they have to be held by the clinician, who exerts the pressure force on the patient’s body. This will be avoided in our design, since the external pressure will be exerted in a controlled way by pneumatic pressure, and also the ergonomics of the device will allow the patient to hold it with the hand on which the pressure will be applied.

The purpose of this work is to design a device with the ability to exert controlled pressure for a set period of time on the thumb with no metal parts, so that it can be used while an MRI scan is being performed to relate brain activity to the level of pain that the patient indicates that they feel.

Several studies relate fibromyalgia to alterations in the brain [27,28], with a significant reduction in most hippocampal fields [29]. The hippocampus presents electrical activity related to the activities that are being carried out at any given moment. This electrical activity can also be observed and recorded in animals while they are performing specific tasks [30]. The desirability of performing an MRI scan to observe the brain response at the moment of suffering painful stimulus has motivated this study. For a better comparison between patients, it is also desired that the stimulus is the same for all, so a scale of forces and application times is established that does not depend on the clinician at the time of application.

Once the MEX technique had been selected, the suitability of the material was checked and, in order to be able to generalise the use of the device, different types of printers were included in the study. The behaviour of different prototypes, based on pieces manufactured in four different 3D printers with six different materials—Polylactic Acid (PLA), Acrylonitrile Butadiene Styrene (ABS), PLA Tough, ABS M30i, Polycarbonate (PC) and Nylon—with a wall thickness of 5 mm and high printing density, were tested. Hardness and surface roughness tests were carried out and the pressure ratio that the device provides in use was checked.

In this paper, Section 2 shows the main goals of the pain meter design and materials and methods for this design. Section 3 concerns the design of the proposed pain meter and, for this purpose, it has been divided into sections covering different aspects. Section 3.1 shows the hardness of the printed parts. The cylinder bore roughness measures obtained for parts made with different printers and materials are shown in Section 3.2. In Section 3.3., the proposed geometry for cylinder and O-Rings and the bore dimensions of the produced cylinders are measured, and in Section 3.4 the final forces obtained in the different prototypes are compared. As a final point, Section 3.5 describes the device control system. In Section 4, the results and discussion of the work are presented, and we end with the conclusions in Section 5.

## 2. Materials and Methods

In order to carry out a specific evaluation of the patient’s brain function, it is desired to design a device without metal elements that can be used inside an MRI machine in contrast to other measuring devices with metallic elements [31]. In this way, a real-time image of the fibromyalgia patient’s brain activity while subjected to measured painful stimulus can be obtained.

The specifications of the system were indicated by a neurologist and the following points were considered:

- fitting inside the MRI scanner;
- possibility of selecting manually from a range of pressures;
- possibility of selecting tests with a predetermined pressure and duration;
- possibility of storing the results obtained with different patients during an evaluation.

**Figure 2:**
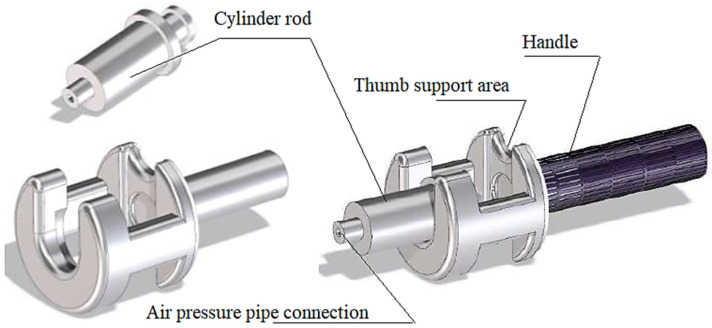
Pain meter handle and cylinder design.

Based on these specifications, several conceptual designs were proposed and finally, a prototype was designed that includes the following subassemblies:

A ***device*** that is to be held by the patient with one hand while in the MRI scanner. It must be light, comfortable to use, and free of metal parts. Therefore, it was decided to design a device to be built by MEX. The device, with a pressure control system, exerts a controlled force on the patient’s thumb over a given period of time. The cylinder rod for pressure on the thumb and the handle design can be seen in Figure 2.

A 4 mm pipe responsible for transporting the pressurised air from the main compressed air installation to the pain meter.

***Pressure regulator***. By means of a current control loop, it will be possible to transmit the pressure that is desired.

***Microcontroller***: used to generate a voltage to produce the current necessary to carry out the control of the pressure regulator. It also serves as a point of connection so that, from a smartphone, tablet or PC, the person in charge of carrying out the test can indicate what pressure is to be used.

***Printed Circuit Board (PCB)***: Contains the signal conditioning circuitry needed to regulate the current of the voltage obtained from the microcontroller.

***Manual regulator***: it is placed between the compressed air intake and the automatic regulator. Its purpose is to limit the air pressure that reaches the automatic regulator and thus avoid possible physical harm to patients, in the event of a failure in the rest of the system.

**Figure 3:**
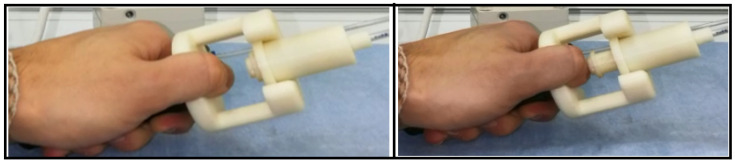
Patient’s thumb position under the piston.

The operation of the pain meter is simple. It has to be held by the patient with one hand while keeping the thumb in the position shown in Figure 3. Once in this position, the patient is placed into the MRI scanner. The doctor decides how much pressure and for how long the force should be exerted, with the patient indicating the level of pain on a scale from no pain to very severe pain.

As can be seen in Figure 2, the handheld device has two MEX-built main sections: on one side, the thumb support area with the handle, and on the other side, the cylinder rod.

**Figure 4:**
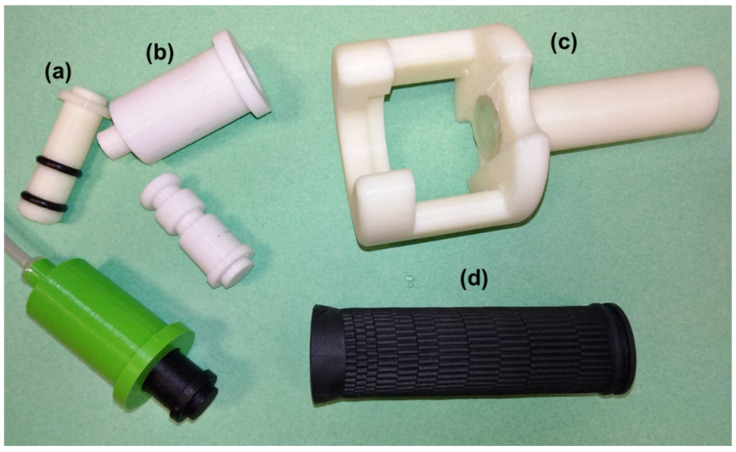
ABS by MEX elements: (**a**) piston; (**b**) cylinder; (**c**) support; (**d**) rubber handle.

The thumb support area (Figure 4c) is ergonomic. Its shape is not easily manufacturable by traditional methods, but MEX technology can be used to produce complex shapes. A rubber commercial handle, cheap and easy to acquire, was added to the base for more comfort (Figure 4d).

The other section designed, the pneumatic cylinder (Figure 4a, piston; Figure 4b, body), must work by providing precision force under the air pressure selected. As it is the most critical part, it must be an independent part that is easy to change if needed, since a loss of precision might easily happen due to the force exerted.

The pieces built by MEX do not have great accuracy nor good superficial quality. Therefore, the most critical MEX-built parts of this device are the cylinder and piston. They are driven by pressurized air, and for their proper functioning it is necessary to obtain a good surface finish of the cylindrical surfaces. For this reason, these particular pieces must be printed with their longitudinal axis vertical, perpendicular to the building plate, so that the finish and dimensions are improved with respect to other orientations [32]. Furthermore, this orientation removes the need for support material on these surfaces; removing the support material would affect the finish and the final dimensions.

Two O-rings (polymer NBR 70) were inserted in the two annular grooves on the outer diameter of the piston to guarantee the precision of fit and avoid air losses.

On the advice of a neurology specialist, the force to be exerted on the thumbnail of the patient should be selectable to an upper limit of around 45 Newton (N). As the normal value of compressed air lines available in hospitals is 6 bar, the internal diameter of the cylinder was set at 16 mm, so that by varying the air pressure from 0 to 3 bar, the force acting on the patient’s thumbnail will vary from 0 to 50 N.

The surface finish and the hardness of the internal surface of the cylindrical hole are important properties that influence the proper functioning of the pneumatic cylinder, as well as the dimensions of the cylindrical hole and the piston and grooves for housing the O-rings.

Six different materials and four different printers were used in this study. Different qualities of printers have been used to validate the functionality of the device. References to both the material used and the printing machine have been made. A number in parentheses has been assigned to each printer for reference in this article. Prusa i3 (Josef Prusa, Prague, Czech Republic), referenced as (1) in Table 1, features an open heating bed and is a low-cost printer, working with PLA. We employed two medium–high range printers with close heating bed and temperature control, the HP Designjet 3D (HP, Palo Alto, CA, USA) (2) working with ABS and the Ultimaker (Ultimaker, Utrecht, The Netherlands) (3) working with PLA Tough. Finally, we utilised the 3D Fortus 450 m Stratasys (Stratasys, Eden Prairie, MI, USA) high-range printer (4) working with PC, ABSM30i and Nylon.

## 3. Results

### 3.1. Measurement of Surface Hardness of Printed Parts

Some articles provide data on the hardness of the materials used in this study [33,34]. However, given the variability depending on the MEX parameters, in this study a Shore D durometer has been used to measure the hardness in all the prototypes.

Table 1 shows the hardness data obtained from the literature and the data resulting from the measurements carried out. Ten Shore D hardness measurements were carried out and the mean value, deviation and variance are shown. All the materials tested had similar Shore D hardness values, between 75 and 80.

### 3.2. Cylinder Bore Surface Roughness

**Figure 5:**
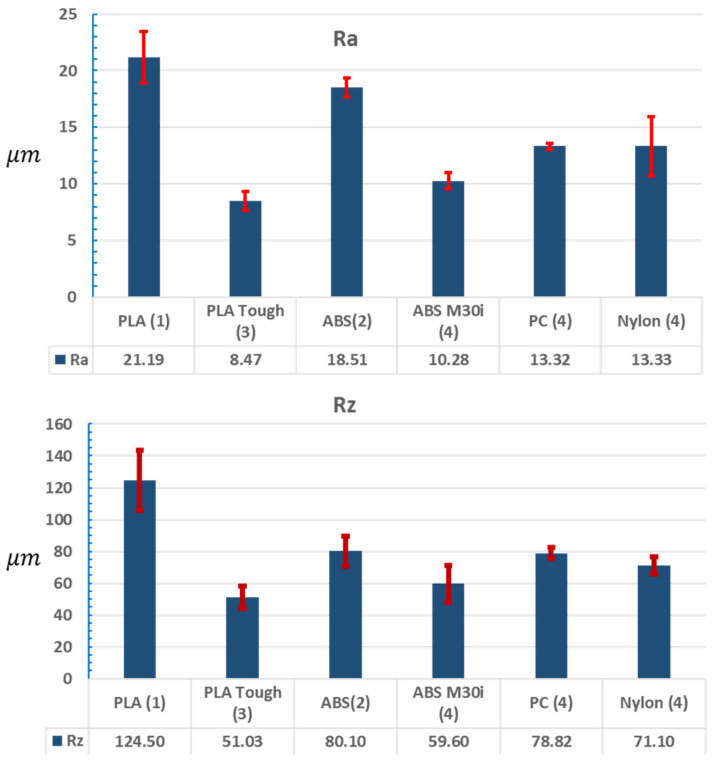
Mean roughness (Ra) and mean roughness depth (Rz) value in μm of the devices made in different materials. The number in parentheses indicates the printer with which the piece was made. The mean value can be seen in blue and the variability obtained for each combination of material and printer in red.

The roughness was measured on the inner face of the cylindrical hole, in four positions separated by 90°, and in the longitudinal direction, coinciding with the displacement of the piston. A MarSurf M300 roughness meter was used (Figure 5). The mean value can be seen in blue and the variability obtained for each combination of material and printer in red.

There are clearly appreciable differences in the roughness of the surfaces, and the best results were obtained for those built in PLA Tough (3) and ABS M30i (4).

### 3.3. Proposed Geometry for Cylinder and O-Rings

**Figure 6:**
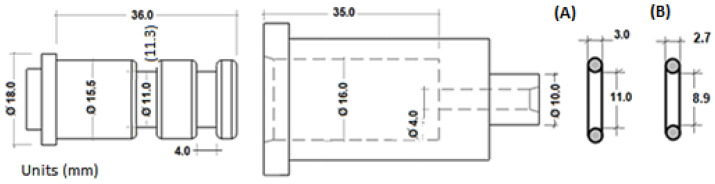
Pneumatic cylinder components, basic dimensions. The dimensions are shown for the two O-rings used, models (A) and (B).

The chosen geometry for the pneumatic cylinder is shown in Figure 6. The diameter of the hole d = 16 mm, and the O-rings used are the most critical element as the quality of their contact determines the functionality of the device. Two types of O-ring were used: O-ring type A) in Figure 6 was mounted on a groove with diameter of 11.0 mm, and O-ring type B) was mounted on a groove with diameter of 11.3 mm. These combinations were used in order to reduce friction and pressure losses. As in other engineering problems, numerous combinations could have been used.

In order to verify the adequacy of the dimensions proposed for the manufacture of the pneumatic cylinder built by MEX without any subsequent mechanisation, all the prototypes were built from the same computer-aided design files (*.stl), with the geometry described in Figure 6.

**Figure 7:**
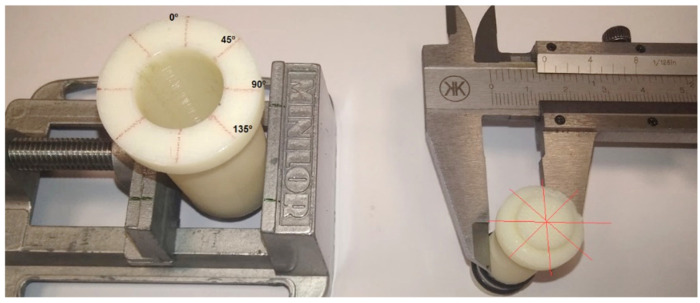
Dimensional tolerance measurement.

**Figure 8:**
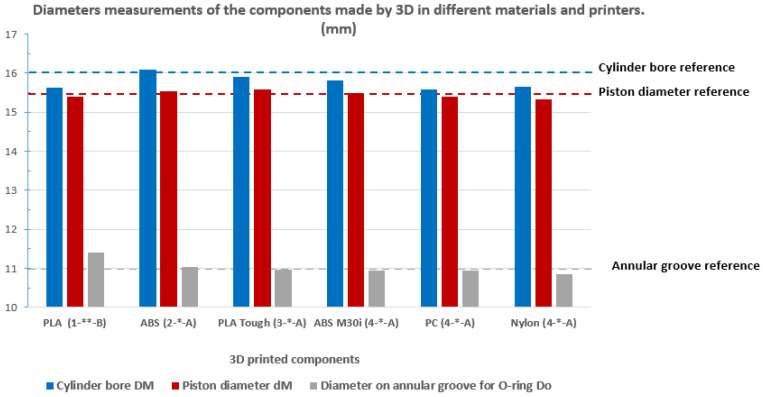
Mean values measured diameters of the piston, cylinder and annular groove printed in different materials with different printers (mm). The measurement specified in the .stl files is indicated, for each component, by a dotted line. The number in brackets refers to the type of 3D printer, the groove diameter in the piston (* 11.0 mm and ** 11.3 mm) and the letters A and B are used for the two O-ring configurations.

After printing the first prototypes, as specified in Table 1 for materials and printers, and trying to assemble them, a great variation in the printers’ tolerances could be seen. Measurements of the diameter of the hole in different positions are shown in Figure 7. The variability of these dimensions can be observed in Figure 8.

The pistons have variations in diameter, and these variations from the proposed dimensions are also found longitudinally for each piston section. The pistons fit without interference in the cylinder holes, except for some prototypes built in PLA (1). Some cases of interference appeared which required the outer surface of the piston to be sanded.

Materials and printers with less dimensional variability are those with the best surface finish. In addition, the best results were obtained for those built in PLA Tough (3) and ABS M30i (4).

### 3.4. Measurement of the Force Exerted by the Pneumatic Cylinder as a Function of the Pressure Exerted for Different Geometries and Materials

**Figure 9:**
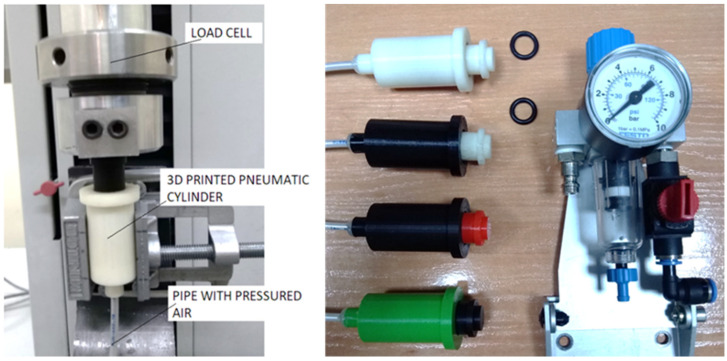
Force measuring arrangement as a function of pressure.

The force exerted on the thumb by the pain meter has been measured on an MTS test machine with a load cell capable of measuring up to 100 N (Figure 9).

**Figure 10:**
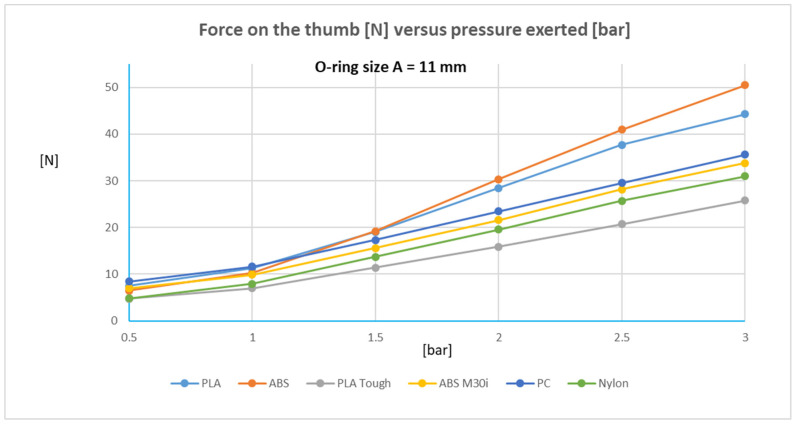
Force on the thumb [N] versus pressure exerted [bar] for the O-ring with configuration A.

**Figure 11:**
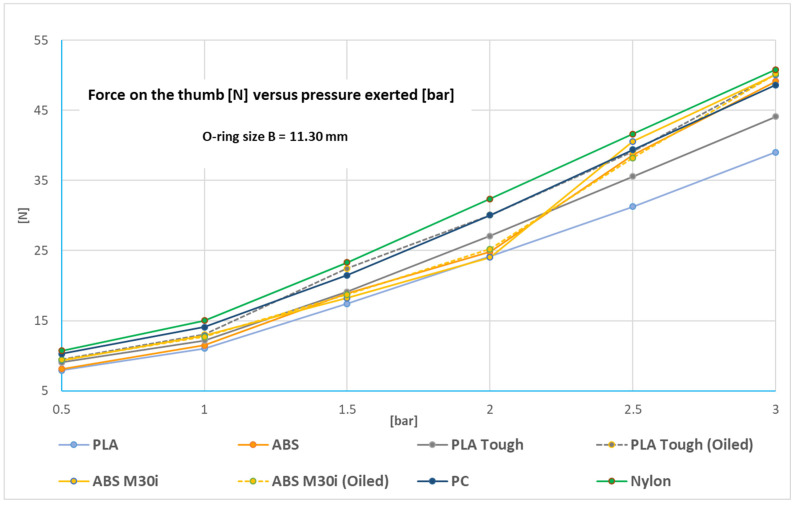
Force on the thumb [N] versus pressure exerted [bar] for the O-ring with configuration B.

Figure 10 (for the O-ring with configuration A) and Figure 11 (for the O-ring with configuration B) show the forces obtained for pressures ranging from 0.5 bar to 3 bar, so that force exerted varies from zero to a maximum around 50 N. To arrive at these designs, it is necessary to take into account the variability in the dimensions of the cylinder hole (see Figure 8) as well as the surface finish of the surface of said hole (roughness, Figure 5), the dimensions of the elastic ring used, and the diameter of the annular groove in the piston.

As PLA Tough and ABS M30i showed the best surface results, a vegetable grease without metal components that can be used inside an MRI scanner was tested in order to check if it improves the device’s performance (see Figure 11, PLA Tough Oiled and ABS M30i oiled). A softer response was observed in PLA Tough, but no significant difference in ABS M30i. The device can be simplified by using it in non-lubricated condition.

The linear trend relating working pressure to exerted force is similar for all materials for the designs with both O-rings (A in Figure 10, B in Figure 11).

**Figure 12:**
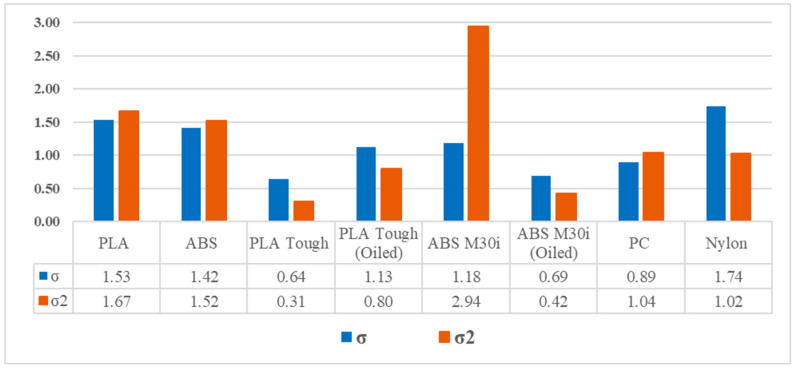
Variance and covariance of the forces obtained for the different materials in which the device was constructed.

Figure 12 shows the variances and covariances of the measurements taken in the case of O-ring B, which allows the selection of the most stable configuration in its results.

Variance and covariance of forces obtained for the different devices are shown in Figure 12. PLA Tough seems to be the best option, providing the most stable results.

The two elastic rings used are suitable for the nominal dimensions of the piston of 15.5 mm diameter, housed in a hole of 16 mm diameter, and with an annular groove of 11 mm diameter in the piston (see Figure 6). In addition, as can be seen in Figure 10 and Figure 11, with the dimensional tolerances of the parts obtained by additive manufacturing, the elastic rings used guarantee repeatability in the forces obtained. This aspect is key in this proposed design. Other combinations may be possible, although the ones selected have been found to be appropriate.

### 3.5. Device Control Elements

Once the device had been designed, it was necessary to establish both electronic control of the device and a control app to facilitate its use.

The industry offers multiple possibilities to make pressure regulations. The SMC model ITV3030 (SMC, Vitoria, Spain) was selected. This model has a control pressure ranging from 0.05 bar to 5 bar with a DC control signal type of 4 mA to 20 mA (https://www.smc.eu/es-es, accessed on 19 December 2024).

This application requires a microcontroller that has the ability to generate PWM waves, Wi-Fi and Bluetooth connectivity, and is small in size. One of the main characteristics that differentiates some microcontrollers from others is connectivity, and not many microcontrollers have it. Some of the devices having this property are the Raspberry Pi, Arduino MKR1000, and LoLin NodeMCU v3 ESP8266; the latter was chosen. It has very high capabilities since it integrates a WiFi module for communication, ADC module for reading analogue signals and generating PWM waves, has 14 pins configurable as input/output, 4 Mbyte memory, supports external power supply voltages ranging from 4.5 V to 9 V and is USB powered. In addition, it has a compact module with reduced dimensions of 30 × 57 mm, which makes it easy to mount almost anywhere near the regulator pressure to be controlled.

**Figure 13:**
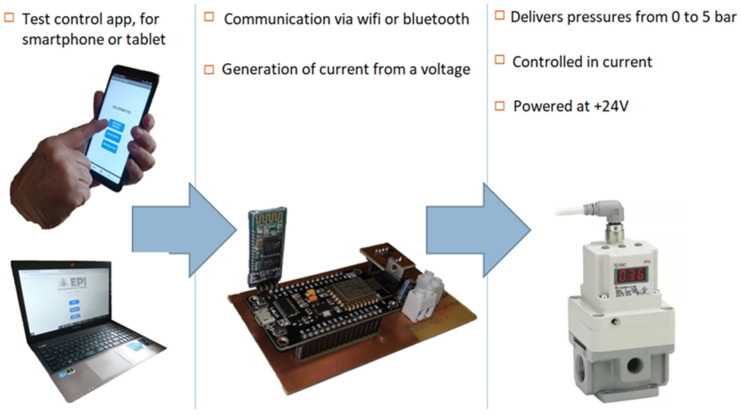
Device control elements.

**Figure 14:**
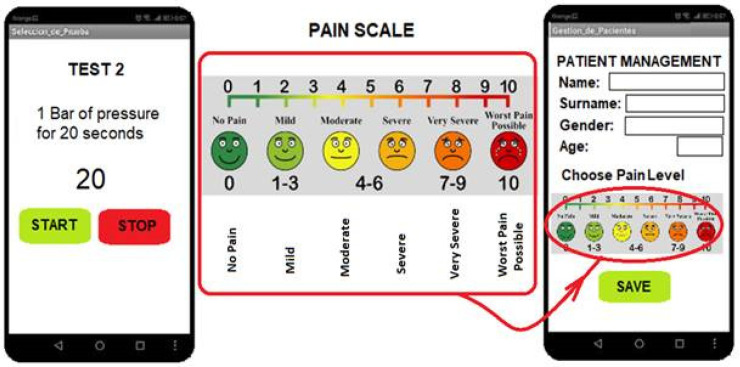
Control app screens for pressure selection and data logging.

The system developed will provide two possibilities for communication between the smartphone or tablet and the electronic control of the device: WiFi and Bluetooth (Figure 13). Figure 14 shows the interface of the control application, where the doctor can evaluate the pain test and the patient’s pain level.

An application has been developed for the processing and storage of patient and pain sensation data. The doctor discusses the scale with the patient at the start of the test and can relate the pressure exerted and the patient’s response to the image shown simultaneously by the MRI scanner. The pain scales used in the diagnosis of fibromyalgia have been widely studied [35,36]. It is necessary to establish a pain scale to study and relate a stimulus, established in intensity and duration, to the patient’s response. In this way, the evolution over time of a patient’s condition can be contrasted or compared with other patients. Figure 14 shows the interface of the application developed for this purpose with the scale commonly used by neurologists in which the patient indicates a degree of pain from no pain to the worst possible pain.

## 4. Discussion

The same printed device was studied on three printers: one low-end, two mid-range and one high-end. Better-quality printers also have a better surface finish (lower Ra), and less dimensional variation. However, one of the mid-range printers (see Table 2) was shown to be fully valid for the manufacturing of this device in any hospital, so a high-performance printer is not necessary.

Its dimensional variations require careful selection of O-ring groove diameter and the size of the O-ring itself in order to achieve a compromise between friction and air leakage that allows repeatability in force tests and the application of the desired force.

The type of stimulus to be used, intensity and time of the pressure exerted on the patient’s thumbnail, requires different forms of study. Some researchers prefer randomized time and intensity series. In other cases, an increase in intensity accompanied by shorter application times is established. Table 2 shows six pre-designed tests relating pressures exerted and application times for a device built by MEX in PLA Tough material, working in both oiled and dry conditions.

Table 2 specifies the types of tests that have been selected for this app. The neurologist can either apply a low pressure and therefore lower force for a longer time or a higher force for a shorter time. Standardizing the tests allows the same times and pressures to be applied to different patients, collecting their pain responses and contrasting them with the images that appear on the MRI.

The roughness of the printed surfaces and the dimensional tolerances of the pneumatic piston, responsible for exerting the force on the thumb, affect the value of the effort and the necessary repeatability, varying from one printer to another and even between different materials in the same printer (Figure 5). Nevertheless, it is possible to find suitable designs, combining the printed parts with adequate dimensions of the piston and the O-rings used, which allow ensuring the reliability of the measures for their use in a pain meter (Table 2).

In addition, the final cost of the prototype represents a much lower total cost than a commercial pain meter that cannot be used simultaneously with an MRI because of its metal elements. This prototype cost includes the device elements obtained by additive manufacturing in ABS, the PCB control printed circuit, two pressure regulators (one manual and one automatic), a variable power supply and the Bluetooth module.

A comparison can be made between the final cost of this prototype and the price of a commercial digital dolorimeter, which is around EUR 2650. The prototype consists of a physical support obtained by additive manufacturing in ABS (EUR 30), a control PCB (EUR 25), two pressure regulators (one manual, EUR 18 and one automatic, EUR 377), a variable power supply (EUR 200) and the Bluetooth module (EUR 5).

The main features of this pain meter system should be highlighted:

- It has the ability to exert variable force on the thumb, defined by a neurologist.
- Ergonomic design, which allows it to be used with both hands (left or right).
- It is a reliable piece of equipment, capable of providing a smooth and continuous movement.
- Repeatability of thumb force at the same air pressure.
- Control system allows the test time and pressure to be preselected.
- The time to be applied for each pressure can be selected by control system.

## 5. Conclusions

At present, the measurement of pain in the diagnosis of fibromyalgia is mainly subjective, which complicates patient treatment. Recent studies show that pain is reflected in brain response observable on an MRI scan.

The present study analyses the procedure of manufacturing, calibration and use of a pain meter without metal parts compatible with simultaneous MRI for better diagnosis.

It has been shown that a pain meter based on a piston actuated with pressurised air and manufactured directly by MEX technology allows application of known forces with proven repeatability. The pain meter designed has been tested with different materials produced by various MEX printers.

This study represents a breakthrough in the use of medical devices anywhere in the world, as the process shown makes it possible to be independent of the type of printer and material used. This is of particular relevance in low-income countries, facilitating the global use of this device.

## Figures and Tables

**Table 1:** Shore D hardness measured on the different prototypes. The material, the printer used and the hardness proposed in the literature are referenced. The number in brackets accompanying the material indicates the printer used in each case.

Material	3D Printer	Shore D *Literature Data*	Shore D Average	Deviation σ	Variance σ^2^
**PLA (1)**	Prusa i3	83	73.50	6.70	44.94
**ABS (2)**	HP Designjet 3D	100	78.00	1.89	3.56
**PLA Tough (3)**	Ultimaker	79	79.00	1.05	1.11
**ABS M30i (4)**	3D Fortus 450 m Stratasys	80	78.70	2.83	8.01
**PC (4)**		80	79.90	2.73	7.43
**Nylon (4)**		80	74.30	1.70	2.90

ABS (Acrylonitrile Butadiene Styrene); PLA (Polylactic Acid); PC (Polycarbonate).

**Table 2:** Working pressure and expected force acting on thumbnail.

Test Number	Air Pressure [bar]	Maximum Time [s]	Force on Thumbnail [N]	
			Prototype: PLA Tough (Oiled)	Prototype: PLA Tough (Dry)
1	0.5	25	9.45	9.05
2	1	20	13.05	12.17
3	1.5	15	22.45	19.13
4	2	10	30.06	27.07
5	2.5	5	39.14	35.58
6	3	5	50.15	44.10
			O-ring B_Dg = 11.30	

## Data Availability

The raw data supporting the conclusions of this article will be made available by the authors on request.
